# Effect of Electric Field on Internal Heat-Flow Characteristics During Evaporation of a Sessile Droplet

**DOI:** 10.3390/mi17060730

**Published:** 2026-06-17

**Authors:** Jiewen Deng, Jiacheng Liu, Li Gan, Guangyuan Kang, Xingyu Shi, Haiting Liu

**Affiliations:** 1 School of Energy and Power Engineering, Northeast Electric Power University, Jilin 132012, China; 20182798@neepu.edu.cn (J.D.); 2202400594@neepu.edu.cn (G.K.); 2202400440@neepu.edu.cn (X.S.); lhtnedu@163.com (H.L.); 2 Henan Jingneng Huazhou Thermal Power Co., Ltd., Anyang 456472, China; ganli2026@163.com

**Keywords:** droplet, evaporation, electric field, multiphysics coupling

## Abstract

Electric-field-enhanced evaporation is an innovative approach to reducing the energy consumption of evaporation processes. To investigate the effect of an electric field on droplet heat-flux characteristics during evaporation, this study numerically simulated the evaporation of pinned sessile droplets on a solid substrate under a parallel-plate electrode configuration. The results show that increasing the plate voltage (from 2 kV to 4 kV) can significantly enhance the surface flow velocity, with the maximum increase reaching 157% in the early stage of evaporation; however, this enhancement diminishes as evaporation progresses. At higher voltages, the internal flow field of the droplet transitions from a single circulation to a double-circulation structure, and the influence of voltage on the droplet’s bulk internal temperature gradient is limited, with the maximum temperature difference remaining below 1 K. When the ambient temperature is below 308.15 K, the electric field enhancement of evaporation is most pronounced: compared with natural (no-field) evaporation, the drying time is reduced by approximately 15.5%. However, when the ambient temperature exceeds 308.15 K, the electric field enhancement decreases markedly. These findings provide a theoretical basis for electric-field-assisted evaporation technologies and inform directions for parameter optimization.

## 1. Introduction

Water evaporation is a fundamental phase-change and mass-transfer process occurring in natural water cycles and across various engineering applications, including industrial separation, energy conversion, and environmental remediation. Conventional evaporation primarily relies on external heating to increase the evaporation rate by raising the liquid temperature, enlarging the liquid–gas interfacial area, or enhancing gas-phase convection. However, because water has a high latent heat of vaporization, conventional thermal evaporation is energy-intensive, particularly in applications such as concentration of high-salinity wastewater [1], seawater desalination [2], humidity control [3], spray drying [4], electronic cooling [5], and microfluidic mass transfer [6]. In these contexts, the trade-off between evaporation efficiency and energy consumption becomes increasingly pronounced. Consequently, the development of low-energy consumption, high-efficiency, and adjustable evaporation enhancement technologies has emerged as a significant research focus within heat and mass transfer, environmental engineering, and the energy and chemical industries. In recent years, advancements in external field regulation technologies have led to the increased application of electric fields in droplet manipulation, enhanced evaporation, and interfacial transport studies, owing to their advantages of non-contact operation, controllability, rapid response, and ease of integration. However, high-voltage electric fields additionally impose strain fields on the system, triggering various electrohydrodynamic phenomena. Researchers such as Digilov [7], Kang [8], Mugele [9], and Celestini and Kirstetter [10] have focused on these phenomena and their applications. Gunji and Washizu [11] investigated self-propelled droplets under the influence of an electric field, introducing the electrostick-slip phenomenon in fixed droplets. In 1976, Japanese scholar Asakawa [12] first demonstrated that external electric fields can influence the evaporation process of droplets. The results indicated that external electric fields can enhance water evaporation, with maximum evaporation efficiency increasing by up to 1.5 times. The degree of influence was contingent upon the properties of the electrodes, the electric fields, and the characteristics of the droplets on the surface. Subsequently, numerous researchers have focused on this area of study [13,14,15,16,17,18,19].

Sharma [20] employed numerical methods to examine how applied electric fields and bulk ion concentration affect the evaporation rate of an electrolyte membrane in contact with a wall. He developed a steady-state model that couples the Poisson–Boltzmann equation for charge distribution with the momentum, energy, and mass conservation equations for the liquid film, and the model also accounts for interaction with moist air flowing over the membrane. Results indicate that volumetric heating from Joule dissipation is non-uniform, producing axial and transverse temperature gradients within the film. Kiyoshi [21] investigated the promoting effect of electrostatic fields on droplet evaporation from heated surfaces, analyzing fundamental electrohydrodynamic (EHD) characteristics and comparing evaporation times of droplets (water, KCL solution, and ethanol) across surface temperatures and field strengths—he discusses enhancement in terms of vapor–liquid interface stability. Lakeh and Molki [22] conducted combined experimental and numerical studies of EHD-enhanced heat transfer in natural convection within circular tubes, reporting that increasing the applied voltage can enhance heat transfer by up to 8.7-fold relative to the no-field case. Havet [23] developed a numerical model of EHD-enhanced forced convection that couples non-isothermal turbulence with the electric field and showed that electrode–ground spacing, conductor radius and arrangement, and applied voltage strongly affect secondary-flow formation and convective heat-transfer enhancement in the channel.

In summary, although electric field control of droplet evaporation has attracted considerable attention, the evolution of internal heat-flow structures during evaporation of pinned (wall-fixed) droplets remains insufficiently understood. Therefore, this paper investigates pinned sessile droplets to clarify how external electric fields influence evaporation kinetics and internal heat-flow dynamics, to identify the dominant mechanisms by which electric fields enhance evaporative heat transfer, and to provide theoretical guidance for the design of electric-field-assisted evaporative heat-exchange devices, microscale thermal management, and precise droplet manipulation.

## 2. Theoretical Basis and Boundary Conditions

**Figure 1:**
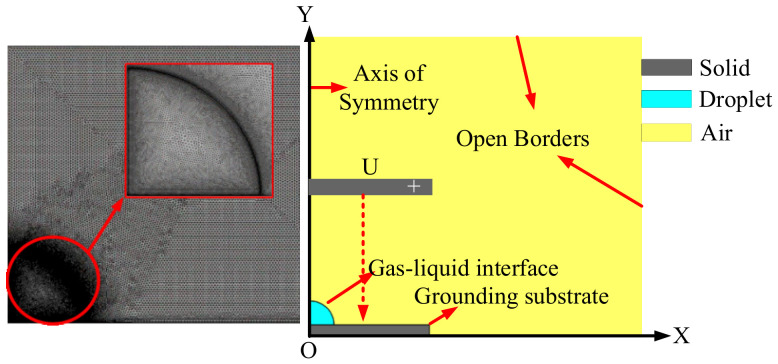
Mesh diagram and model boundary conditions.

**Figure 2:**
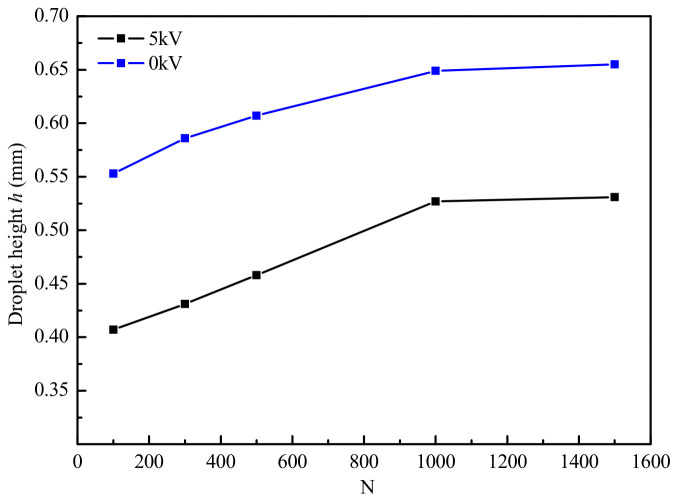
Mesh independence verification.

Figure 1 shows the mesh diagram and the schematic diagram of the computational model for the evaporation of a fixed droplet with radius *r*_0_ in a direct-current (DC) electrostatic field. A two-dimensional symmetric model was employed for the simulations. The outer boundary of the computational domain was treated as open, and a Cartesian coordinate system was used. The moving mesh method was employed to simulate flow in the liquid–gas two-phase domain and to track the liquid–gas interface by coupling the moving mesh module with the laminar two-phase flow module. Within the arbitrary Lagrangian–Eulerian (ALE) framework, the interface motion was synchronized with the mesh displacement, maintaining mesh adherence at the moving phase boundary. Compared with alternative interface-capturing approaches, such as the level-set and phase-field methods, the deforming-mesh approach yielded more accurate interface positions while requiring less computational cost. The initial radius of the droplet is R, the height of the stainless-steel substrate is 1 mm, the width is 10R, and the electrode spacing is 10R. The minimum grid cell in the droplet region is R/N, the grid size at the liquid surface, the centerline of symmetry, and the contact position between the droplet and the wall are *R/N*, the maximum grid cell is 5 times the minimum grid cell, and the maximum cell growth rate is 1.05. In this paper, COMSOL Multiphysics 6.0 software is used for numerical simulation. To verify the mesh independence, the height of the droplet after 600 s of evaporation was obtained under different mesh sizes, as shown in Figure 2. The results show that when *N* is 1000 and 1500 the calculated results are very close, with an error of less than 1% between them. To save computational resources, *N* = 1000 is selected.

The evaporation of a wall droplet under an electric field is a highly complex multiphysics process. To achieve multiphysics coupled calculations with the rationality of the model, the main assumptions of the numerical model are as follows:

(1) The flow in the liquid and gas regions is considered incompressible. Due to the size of the droplet, its shape is not significantly affected by gravity; therefore, the initial shape of the droplet is assumed to be a spherical cap.

(2) Since the air breakdown field strength between parallel plates is about 3 MV/m [24], the electric field strength of the voltage range selected in this paper does not reach the breakdown field strength when the plate spacing is 5 mm; therefore, the effect of ionic wind on droplets is ignored, and the transfer or neutralization of the droplet charge is ignored.

(3) During the droplet evaporation, the droplet radius remains unchanged, and the evaporation mode is simplified to the CCR mode.

(4) Since this paper mainly investigates the effect of the electric field on the internal motion of the droplet, and the temperature variation is small, the Marang effect caused by the temperature difference generated inside the droplet during the evaporation process is ignored.

Electrostatic equation:

In this paper, the electric field strength *E* is set to be irrotational, which can be described by the following equation:

(1) ∇×E=0,

where the electric field strength can be described by the electric potential *V*:

(2) E=−∇V.

The electric displacement field *D_1_* is expressed as follows:

(3) $$D_{1}=\varepsilon_{0}\varepsilon_{r}E,$$

where *ε_0_* and *ε_r_* are the permittivity and relative permittivity of the vacuum, respectively.

The electrostatic field satisfies Poisson’s equation [25]:

(4) $$\nabla \cdot D_{1}=\nabla \cdot (\varepsilon_{0}\varepsilon_{r}E)=\rho_{v},$$

where *ρ_v_* is the volume charge density, which satisfies the charge transport equation:

(5) $$\frac{\partial \rho_{v}}{\partial t}+\nabla \cdot (\rho_{v}u_{c})=\nabla \cdot (\tau \nabla V).$$

Here, τ denotes the electrical conductivity. Because the partial differential equations were formulated in the grid coordinate system, the fluid velocity *u* in the governing equations corresponds to the velocity expressed in the grid (mesh) frame [26,27]. Owing to droplet deformation and the non-uniform distribution of surface charge, the magnitude of the electric field force on the droplet depended on the surface charge density, the separation between the liquid surface and the electrode, and other parameters. The net Maxwell stress acting on the droplet surface can be expressed by the surface integral:

(6) $$F_{e}={\int_{S}T}_{i}^{E}dS,$$

where *S* is the droplet surface area and *T_i_^E^* is the Maxwell stress tensor.

The electric field force subjected to a droplet under the action of a uniform electric field can be added to the source term in Navier–Stokes Equation (11) in the form of “volume force”, and the Maxwell stress caused by the electric field is added to the stress equilibrium at the interface, and the equilibrium equation is as follows:

(7) $$\vec{n}\cdot \left(T_{c}^{H}-T_{d}^{H}\right)+\vec{n}\cdot \left(T_{c}^{E}-T_{d}^{E}\right)=\sigma (\nabla_{s}\cdot \vec{n})\vec{n}-\nabla_{s}\sigma ,$$

where the subscripts *d* and *c* represent the characteristics of the dispersed phase and the continuous phase, respectively. *σ* is the surface tension, and the electrical stress at the interface causing the droplet deformation is expressed in a weak form by introducing a test function. *T_i_^E^* is the Maxwell stress tensor and *T_i_^E^* is the hydrodynamic stress tensor [28]:

(8) $$T_{i}^{E}=\varepsilon_{i}(EE-\frac{1}{2}(E\cdot E)I),$$

(9) $$T_{i}^{H}=-P_{i}I+\mu_{i}(\nabla u+{\left(\nabla u\right)}^{T}).$$

Incompressible fluid continuity equation and momentum equation:

(10) ∇·u=0,

(11) $$\rho \frac{\partial u}{\partial t}+\rho (u_{c}\cdot \nabla )u=\nabla \cdot [-PI+\mu (\nabla u+{\left(\nabla u\right)}^{T})]+F.$$

In the formula, *u* is the fluid velocity, *u_c_* is the material diffusion velocity, *P* is the fluid pressure, *ρ* is the fluid density, *μ* is the fluid viscosity, *I* is the identity matrix, and *F* is the source term. The evaporation process of droplets in the electric field environment is studied in this paper; therefore, the source term *F* in the droplet region is the sum of the droplet gravity and the electric field force *F_e_* on the droplets.

Heat transfer equation in liquid and gas phases:

(12) $$\rho C_{p}\frac{\partial T}{\partial t}+\rho C_{p}(u_{c}\cdot \nabla )T=\nabla \cdot (k\nabla T).$$

Transport equation of various substances and steam:

(13) $$\frac{\partial c}{\partial t}+(u_{c}\cdot \nabla )c=\nabla \cdot (D\nabla c),$$

where *c* is the steam concentration and *C_p_* is the specific heat capacity of the substance.

The saturated vapor pressure is obtained according to the Antoine equation:

(14) $$P_{sat}=10^{\left[A-B/(C+T)\right]}$$

Among them, *A*, *B*, and *C* adopt the constants in [29], and take the values of 8.10765, 1750.286, and 235, respectively. According to the ideal gas law, the saturated vapor concentration can be obtained as follows:

(15) $$c_{sat}=\frac{M_{vapor}P_{sat}}{R_{q}T}.$$

On the droplet surface, the evaporated mass flux of water vapor can be expressed as:

(16) $$J\;={\;M}_{\mathrm{vapor}}\vec{n}\cdot \left(-D\cdot \nabla c\right),$$

where $\vec{n}$ is the normal direction of the droplet surface, and *D* is the diffusion coefficient of water vapor in air. To simplify the calculation, this paper assumes that the diffusion coefficient of water vapor in air is constant, which is taken as 2.85 × 10^−5^ m^2^/s.

Governing equation for moving mesh transformation:

(17) $$\left\{\begin{array}{l} \frac{\partial^{2}}{\partial r^{2}}\frac{\partial R_{m}}{\partial t}+\frac{\partial^{2}}{\partial z^{2}}\frac{\partial R_{m}}{\partial t}=0\\ \frac{\partial^{2}}{\partial r^{2}}\frac{\partial Z_{m}}{\partial t}+\frac{\partial^{2}}{\partial z^{2}}\frac{\partial Z_{m}}{\partial t}=0 \end{array}\right..$$

To ensure that the droplet remains in a pinned state during evaporation, the displacement of the three-phase contact line is restricted.

**Figure 3:**
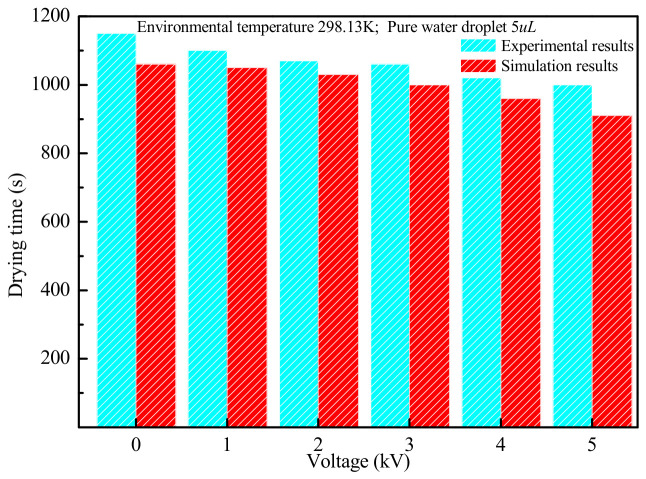
Experimental and numerical results of droplet drying time.

Due to the extremely slow internal flow process of the droplet during evaporation, it is difficult to measure using experimental methods. To verify the rationality of the numerical method constructed in this paper, the droplet drying time is currently selected as the basis for comparison. Figure 3 shows experimental and numerical results of droplet drying time. The results indicate that for a 5 μL pure water droplet, under the conditions of an ambient temperature of 25 °C and a plate spacing of 5 mm, the evaporation time decreases as inter-plate voltage increases. The variation trend of the numerical results is basically consistent with that of the experimental results, and the maximum error between the two is less than 10%, which indicates that the numerical model constructed in this paper is reasonably valid.

## 3. Results and Discussion

### 3.1. Influence of Electric Field Intensity on Heat-Flow Characteristics in Droplets

From Equation (1), the electric field strength *E* scales linearly with the applied voltage *V* and inversely with the electrode spacing *d* (*E ≈ V/d*). To investigate the effect of electric field strength on droplet evaporation, we analyzed internal heat-flow characteristics at different applied voltages while fixing the electrode spacing at 5 mm.

**Figure 4:**
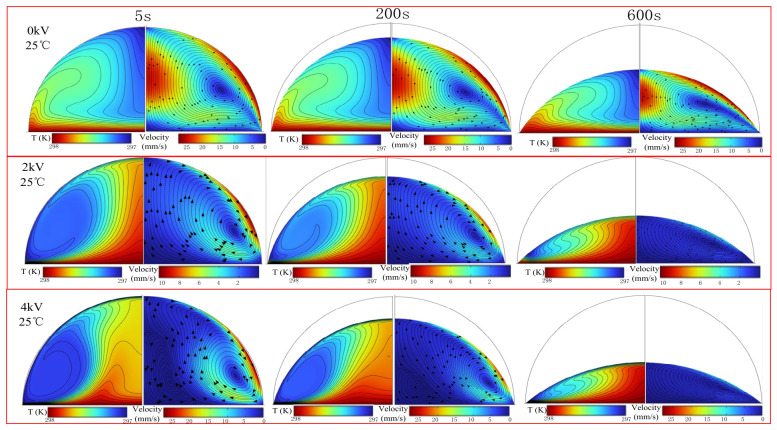
Temperature and velocity distribution in the droplet region under different electrode voltage conditions.

We compared parallel-plate electric field cases with plate voltages of 0, 2 kV, and 4 kV. Figure 4 shows temperature and velocity distributions in the droplet region for these voltage conditions. The results indicate that in the parallel-plate configuration, under zero applied voltage the droplet exhibits a single internal recirculation cell: the fluid travels along the droplet apex on the liquid surface and then returns from the central region toward the electrode. When a 2 kV voltage is applied, the internal flow direction reverses relative to the zero-voltage case. At 4 kV, two distinct internal circulation zones form within the droplet. Consequently, the central region exhibits a steep temperature gradient with a spatial distribution opposite to that under no-field conditions, indicating that the parallel-plate electric field can modulate heat and mass transfer in the droplet by altering internal flow patterns.

**Figure 5:**
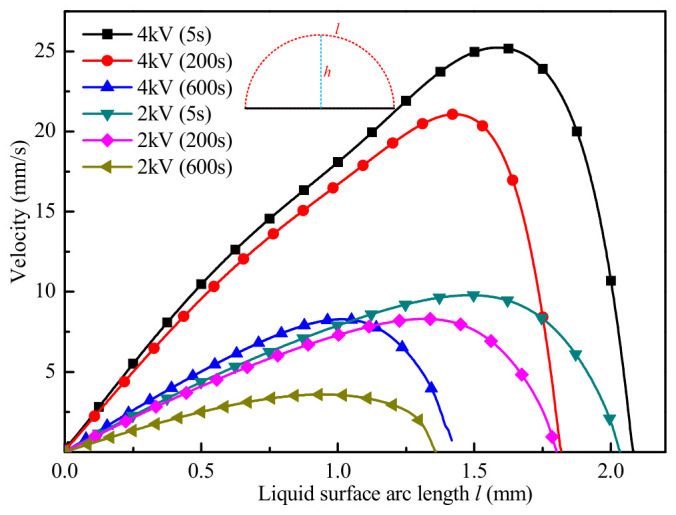
Distribution of liquid surface velocity.

Figure 5 shows the distribution of liquid surface velocity (from the top of the droplet to the three-phase contact line of the droplet) under different voltage conditions. The results show that in the plate–plate electric field, from the top of the droplet to the three-phase contact line, the liquid surface velocity first increases and then decreases, and the high-flow-velocity position of the liquid surface gradually shifts to the top of the droplet with the evaporation. When the droplet evaporation was carried out for 5 s and the plate voltage was 2 kV, the peak flow velocity of the liquid surface was 9.8 mm/s and the average flow velocity was 6.2 mm/s. The plate voltage is increased to 4 kV, the peak flow velocity of the liquid surface is increased to 25.2 mm/s, an increase of 157%, and the average flow velocity is increased to 15.5 mm/s, an increase of 150%. When the plate voltage increases from 2 kV to 4 kV for 200 s, the peak flow velocity of the liquid surface increases from 8.3 mm/s to 21.1 mm/s, with an increase of 154%, and the average flow velocity increases from 5.3 mm/s to 12.9 mm/s, with an increase of 143%. When evaporation reaches 600 s, when the plate voltage increases from 2 kV to 4 kV, the peak flow velocity of the liquid surface increases from 3.6 mm/s to 8.3 mm/s, an increase of 131%, and the average flow velocity increases from 2.4 mm/s to 5.2 mm/s, an increase of 53.8%. It can be seen that increasing the plate voltage can increase the liquid surface flow velocity, but with the progress of evaporation, the strengthening effect of voltage on the liquid surface flow velocity is weakened.

**Figure 6:**
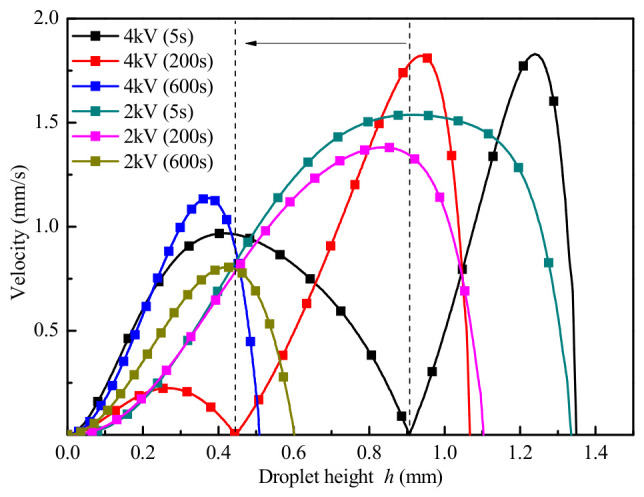
Velocity distribution along the vertical line in the droplet.

Figure 6 shows the velocity distribution along the vertical centerline from the substrate center to the droplet apex. The results show that at 2 kV the velocity along this line initially increases from the substrate toward the apex and then decreases. At 4 kV, two velocity peaks appear along the centerline during evaporation; at later times, these peaks merge into a single peak. This behavior indicates the formation of two internal circulation zones in the droplet center, with a low-speed region at the junction between them. In the later stage the two zones coalesce into one circulation cell (this behavior is discussed below). Quantitatively, at t = 5 s, increasing the plate voltage from 2 kV to 4 kV raises the peak centerline velocity from 1.5 mm/s to 1.8 mm/s (≈ +20%), while the average centerline velocity falls from 1.1 mm/s to 0.8 mm/s (≈ −27.3%). At t = 200 s, the peak increases from 1.4 mm/s to 1.8 mm/s (≈ +28.6%), while the average decreases from 0.9 mm/s to 0.8 mm/s (≈ −11.1%). At t = 600 s, the peak rises from 0.8 mm/s to 1.1 mm/s (≈ +37.5%) and the average increases from 0.5 mm/s to 0.7 mm/s (≈ +40%). Thus, in the early evaporation stage, higher plate voltage increases the maximum centerline velocity but reduces the mean centerline velocity. This arises because higher voltage can split the center flow into two counter-rotating zones, lowering the overall mean velocity along the centerline. The centerline velocity is, therefore, less sensitive to plate voltage than the surface flow. Consistent with Figure 5, increasing the electrode voltage primarily enhances the liquid-surface flow velocity.

**Figure 7:**
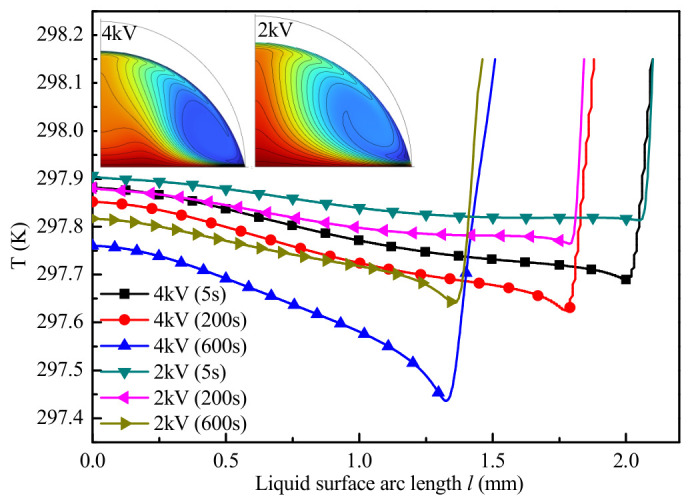
Distribution of liquid level temperature.

**Figure 8:**
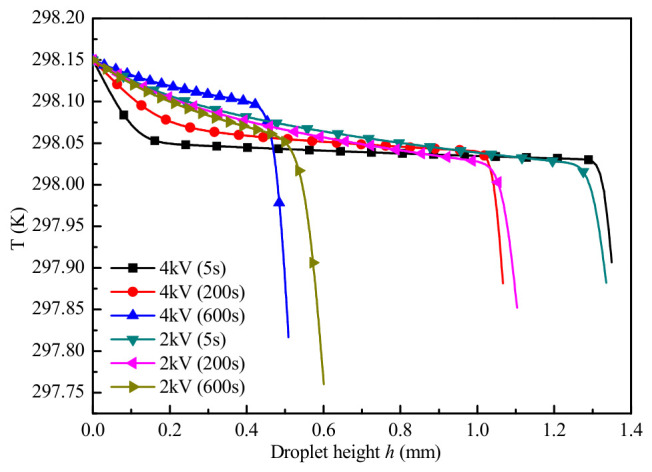
Temperature distribution along the vertical line in the droplet.

Figure 7 shows the temperature distribution along the liquid surface. The temperature along the surface decreases slowly from the droplet apex toward the contact line, then increases sharply near the substrate. As plate voltage increases, the temperature difference between the apex and the contact line grows, and this difference becomes more pronounced as evaporation proceeds. Figure 8 shows the temperature profile along the central vertical line from the substrate center to the droplet apex. In this region the temperature falls gradually from the substrate toward the apex, with a rapid drop near the apex. At low voltage the apex temperature is lower, while the droplet height decreases only slightly over time. Across all cases the maximum temperature difference inside the droplet remains below 1 K, indicating that the plate–plate electric field has only a minor effect on the internal temperature gradient. According to Formulas (14) and (15), when the overall temperature difference is less than 1 K, the temperature gradient can cause fluctuations of approximately ±1% in local saturated vapor pressure and local evaporation flux through the Antoine equation, with negligible impact.

**Figure 9:**
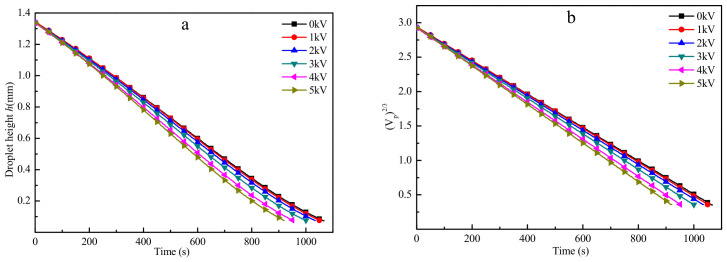
Variation of droplet parameters over time. (**a**) The height of the droplet changes with time. (**b**) The 2/3 power of the droplet volume varies with time.

**Figure 10:**
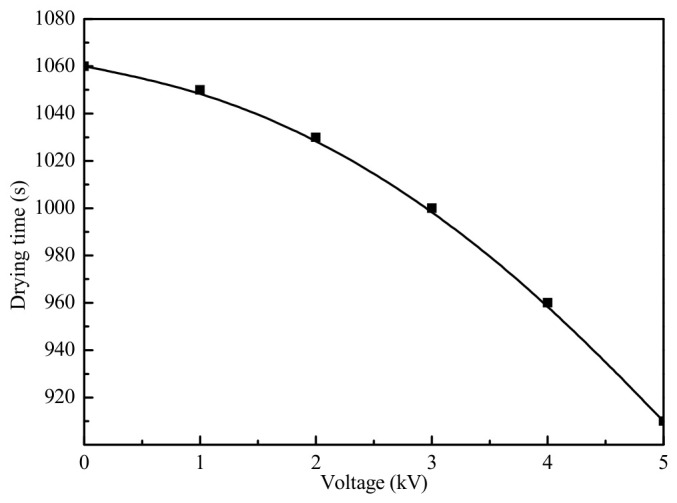
Droplet evaporation drying time at different voltages.

Figure 9a,b show the variation of the droplet height and the 2/3 power of the droplet with evaporation time, respectively. The results in Figure 9a indicate that in the plate–plate electric field, the higher the voltage of the upper electrode plate, the more significantly the droplet height decreases with time. Under different voltage conditions, the droplet height exhibits a nearly linear relationship with time for most of the evaporation period, but it is not a linear variation. This is because the initial contact angle of the droplet is 90°, and the relationship between the droplet volume V and height *h* approximately satisfies *V* ∝ *h^2^* over a wide range, while the nonlinear characteristics become significant at the end of evaporation (as *h* approaches 0). The results in Figure 9b show that during the droplet process, the 2/3 power of the droplet volume is linearly related to time, which is consistent with the classical droplet evaporation law [30]. Experimental conditions were initial droplet volume 5 μL, ambient temperature 298.15 K, and contact angle 90°. Evaporation time (defined as the time to lose 95% of the initial volume) for natural evaporation was 1060 s. With the upper-plate voltage set to 1, 2, 3, 4, and 5 kV, the evaporation times were 1050, 1030, 1000, 960, and 910 s, as shown in Figure 10. Respectively, these results show a nonlinear but monotonic decrease of evaporation time with increasing voltage, and the reduction becomes more pronounced at higher voltages.

### 3.2. Effect of Temperature on Droplet Evaporation Under Electric Field

**Figure 11:**
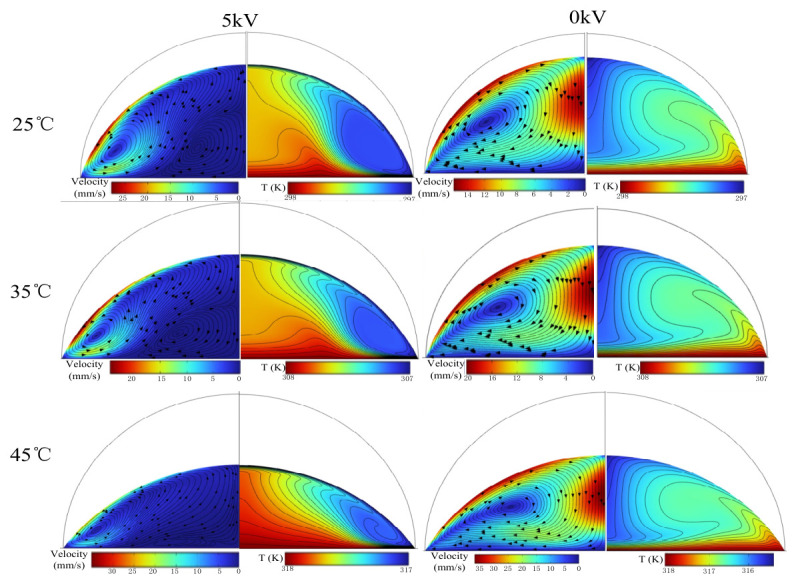
Temperature and velocity distribution inside a droplet at different temperatures.

We observed that the applied electric field produced little additional enhancement of evaporation at elevated ambient temperatures. To investigate this, we compared the temperature and velocity fields inside the droplet under different ambient temperatures. Figure 11 shows the temperature and velocity fields at t = 200 s. Without an applied field, raising the ambient temperature does not appreciably change the location of the internal recirculation cell. With the upper plate at 5 kV, the velocity field exhibits two circulation zones, the stronger of which lies near the three-phase contact line. As ambient temperature increases and evaporation accelerates, the two zones begin to merge into a single circulation cell; the cell center shifts downward, moving closer to the contact line.

**Figure 12:**
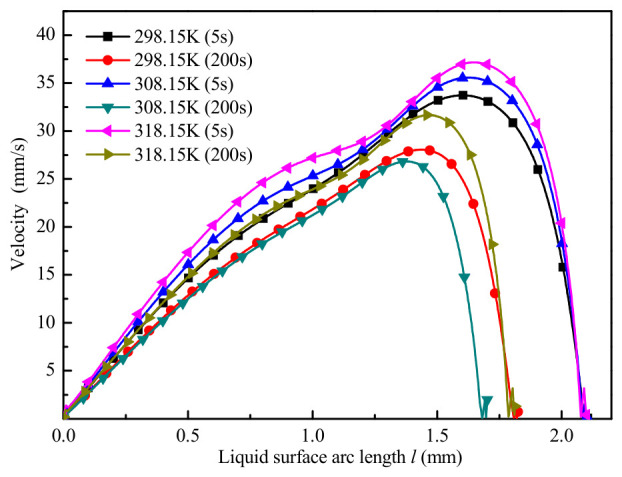
Velocity distribution of the liquid surface.

**Figure 13:**
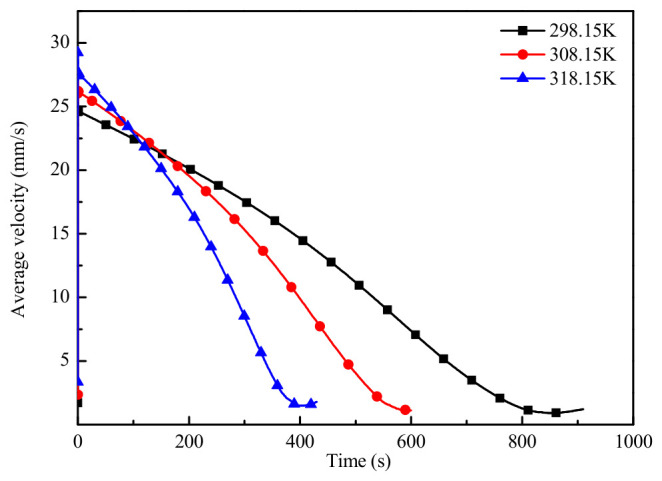
Variation of average surface velocity with time.

**Figure 14:**
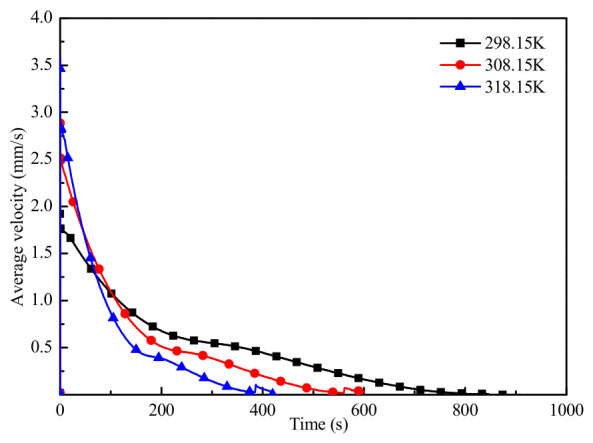
The average velocity of the liquid droplet centerline at different times.

**Figure 15:**
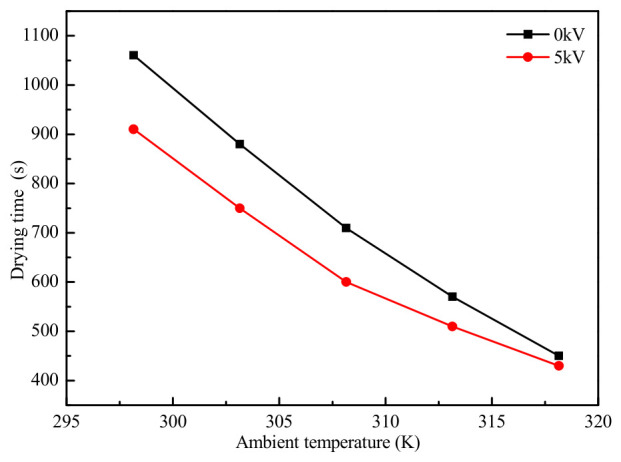
Drying time of droplets at different temperatures.

Figure 12 shows the distribution of liquid surface velocity at 5 s and 200 s. The results show that when the temperature rises, the peak values of liquid surface velocity all increase. At 5 s, when the ambient temperature is 298.15 K, 308.15 K, and 318.15 K, the peak liquid surface velocity is 33.7 mm/s, 35.6 mm/s, and 37.2 mm/s, respectively, and the average liquid surface velocity is 20.8 mm/s, 22.1 mm/s, and 23.4 mm/s, respectively. It can be seen that the current peak and average liquid surface velocity both increase with the increase of temperature. At 200 s, when the ambient temperature is 298.15 K, 308.15 K, and 318.15 K, the peak velocity of the liquid surface is 28.1 mm/s, 26.8 mm/s, and 31.7 mm/s, respectively, and the average velocity of the liquid surface is 17 mm/s, 16 mm/s, and 19 mm/s, respectively. At present, the peak velocity and average velocity of the liquid surface decrease first and then rise with the increase of temperature. Figure 13 shows the change of the average velocity of the liquid surface with time. The results show that in the plate–plate electric field, although increasing the ambient temperature can increase the flow rate of the liquid surface at the initial stage of evaporation, after 150 s, the higher the ambient temperature, the average flow rate of the liquid surface decreases obviously. The current electric field cannot increase the flow rate of the droplet surface to achieve enhanced evaporation. Figure 14 shows the average flow velocity of the droplet centerline at different times. The results show that under the action of the electric field in the high-temperature process, the average temperature drops obviously at the same time. When evaporation proceeds for 100 s, the average flow velocity in the droplet center region begins to decrease with the increase of temperature, and this turning phenomenon occurs earlier than the liquid surface. Figure 15 shows the comparison of drip evaporation time with and without the electric field at different temperatures. Compared with natural evaporation, when the plate voltage is 5 kV, the ambient temperatures are 298.15 K, 303.15 K, 308.15 K, 313.15 K, and 318.15 K, and the electric field shortens the evaporation time by 150 s, 130 s, 110 s, 60 s, and 20 s, respectively. Compared with natural evaporation, the shortened evaporation time is 14.2%, 14.7%, 15.5%, 10.5%, and 4.7%, respectively. It can be seen that the electric field enhances the evaporation effect more obviously when the environment is 308.15 K, and the electric field enhances the evaporation effect obviously after exceeding this value.

## 4. Conclusions

By constructing a coupled thermal-mass model of droplet evaporation under the action of the electric field, the influence of electric field parameters and ambient temperature differences on the heat-flow characteristics inside droplets is studied in this paper, which provides references for controllable evaporation, fine thermal management, and microscale fluid manipulation. The main conclusions are as follows:

(1) Increasing the parallel-plate voltage from 2 kV to 4 kV substantially increased the liquid-surface flow: at the initial stage of evaporation the peak surface velocity increased by 157% and the mean surface velocity by 150% (relative to 2 kV). As evaporation progressed, the enhancement weakened; by t = 600 s, the velocity increases had fallen to ≈131% (relative to 2 kV).

(2) High voltage will change the flow field structure inside the droplet, so that the inside of the droplet will change from a single circulation to a double-circulation region. However, the effect of the electric field on the temperature gradient inside the droplet was not significant, and the maximum temperature difference was less than 1 K.

(3) The electric field enhancement was strongest at or below 308.15 K. At 5 kV, the evaporation time was reduced by up to 15.5% (largest reduction observed at 308.15 K) compared with natural evaporation. Above 308.15 K, the effect weakened markedly; for example, at 318.15 K the evaporation time was reduced by only 4.7% under the same 5 kV field.

## Data Availability

Data are contained within the article.
